# DNA-based immunotherapy for cancer: *In vivo* approaches for recalcitrant targets

**DOI:** 10.1016/j.ymthe.2025.04.008

**Published:** 2025-04-09

**Authors:** Pratik S. Bhojnagarwala, Joshua Jose, Shushu Zhao, David B. Weiner

**Affiliations:** 1 Vaccine and Immunotherapy Center, The Wistar Institute, 3601 Spruce Street, Philadelphia, PA, USA

**Keywords:** cancer, immunotherapy, vaccine, DNA vaccine, neoantigens, clinical trials

## Abstract

Immunotherapy has revolutionized cancer treatment and complements traditional therapies, including surgery, chemotherapy, radiation therapy, and targeted therapies. Immunotherapy redirects the patient’s immune system against tumors via several immune-mediated approaches. Over the past few years, therapeutic immunization, which enable the patient’s T cells to better recognize and kill tumors, have been increasingly tested in the clinic, with several approaches demonstrating treatment improvements. There has been a renewed interest in cancer vaccines due to advances in tumor antigen identification, immune response optimization, novel adjuvants, next-generation vaccine delivery platforms, and antigen designs. The COVID-19 pandemic accelerated progress in nucleic acid-based vaccine manufacturing, which spurred broader interest in mRNA or plasmid platforms. Enhanced DNA vaccine designs, including optimized leader sequences and RNA and codon optimizations, improved formulations, and delivery via adaptive electroporation using stereotactic intramuscular/intradermal methods have improved T cell responses to plasmid-delivered tumor antigens. Additionally, advancements for direct *in vivo* delivery of DNA-encoded monospecific/bispecific antibodies offer novel tumor-targeting strategies. This review summarizes the recent clinical data for therapeutic cancer vaccines utilizing the DNA platform, including vaccines targeting common tumor-associated and viral antigens and neoantigen vaccines using nucleic acid technologies. We also summarize preclinical data using DNA-launched monoclonal/bispecific antibodies, underscoring their potential as a novel cancer therapy tool.

## Graphical abstract

**Figure:**
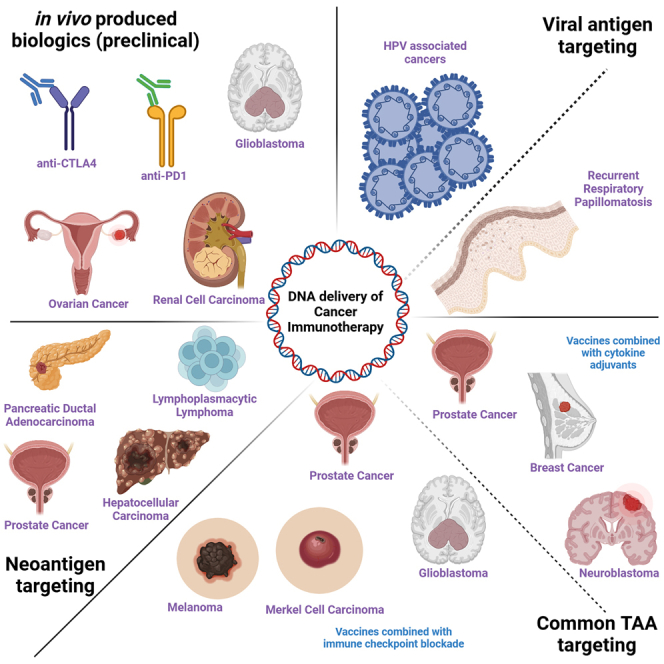

## Teaser

Bhojnagarwala and colleagues review recent clinical trials testing therapeutic vaccines for cancer using the DNA platform. They summarize vaccines targeting HPV-derived antigens, common tumor-associated antigens, and neoantigens. They analyze the impact of adjuvants and ICB on clinical outcomes. Finally, they summarize DNA-launched monospecific/bispecific antibodies as a novel tool for cancer therapy.

## Introduction

**Figure 1:**
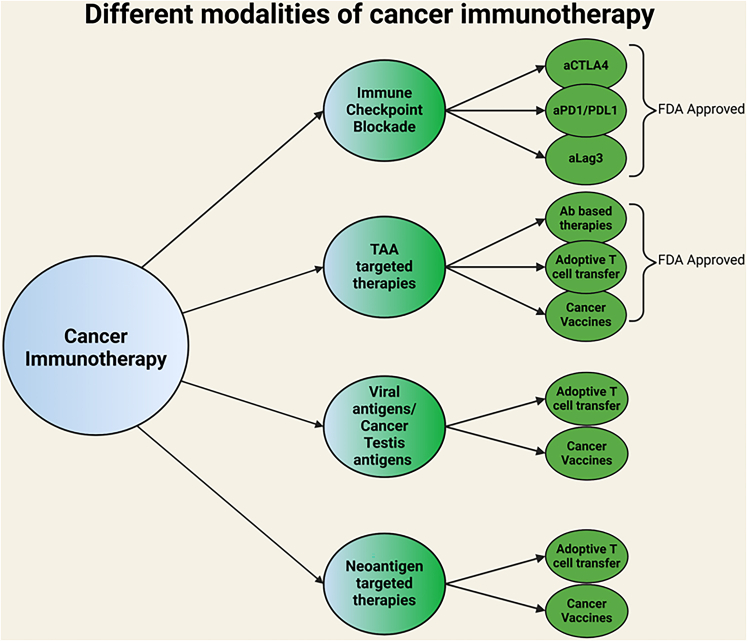
The different modalities of cancer immunotherapy a, anti; Ab, antibody; TAA, tumor-associated antigen.

Although there have been significant advances in treatment, cancer remains among the most significant health concerns, resulting in almost 700,000 deaths per year in the United States, with millions of additional deaths globally.^1^ Building on now-standard methods of cancer treatment, including surgery, chemotherapy, and radiation therapy, new approaches such as targeted therapies and now immunotherapies, are further improving patient outcomes. Immunotherapies redirect the patient’s immune system to better recognize and control tumors.^2^ There can be several different forms of immunotherapy (Figure 1), each with their distinct mechanism of action allowing focused targeting of diverse cancers.

**Figure 2:**
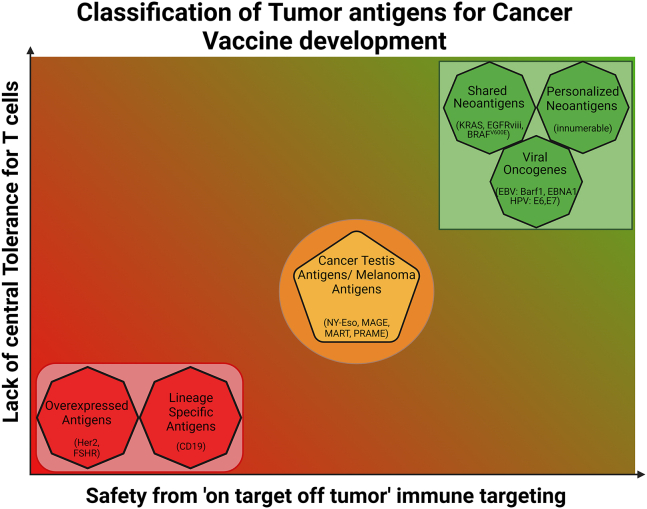
Classification of tumor antigens for cancer vaccines based on lack of central tolerance for T cells and specificity of expression on tumors Examples in parentheses are representative and not an exhaustive list.

Therapeutic cancer vaccines build on the exceptional impact generated by traditional vaccination methods for infectious disease. Vaccines against infectious diseases, including smallpox, influenza, measles, mumps, rubella, rotavirus, polio, hepatitis B virus, human papillomavirus (HPV), diphtheria, tetanus, pertussis, and, recently, COVID-19 vaccines, among others, have been in wide use, some for more than a century. Overall, they are well tolerated, highly effective, and exceptionally safe in the general population. Their focus is to show antigens from the pathogen to the vaccinee in a controlled way to have the host’s immune system to engage adaptive immunity. Adaptive immunity is the inducible arm of the immune system, which includes antibodies and T cells that uniquely recognize the pathogen and allow for a wall of immunity to exist in individuals before they encounter the pathogen for the first time. The most impactful of these prevent devastating pathogens from causing disease in the general population, resulting in enormous improvements in patients’ morbidity and mortality. These types of vaccines are given to healthy individuals to induce preexisting protection, mostly antibody based, but there is a growing recognition of the importance in T cell immunity for longer-lasting disease control and infection clearance. In contrast, therapeutic cancer vaccines/therapies face an even greater challenge in that they are designed to redirect patient immunity to target pathogenic precancerous or cancerous cells to regress or eliminate tumors (i.e., established or at least minimal disease). The antigenic targets of cancer vaccines can be derived from several sources such as viral oncogenes, tumor-associated antigens (TAAs), neoantigens, and lineage markers.^3^ In addition to incorporating antibody-like responses for targeting tumor antigens expressed on the cell surface, the CD8^+^, or so-called killer T cells and natural killer (NK) cells, are considered important effector cells to clear cancer cells, especially those whose tumor antigens are not expressed on the cell surface. Some key considerations for choosing vaccine target antigens include the specificity of tumor expression and T cell central tolerance (the process by which autoreactive lymphocytes are eliminated and prevented from entering the periphery^4^), which could recognize these antigens (Figure 2). Designs for the antigen delivery that can engage the unique CD8^+^ T cell lineage, providing help to expand the immune response in the immunosuppressive tumor microenvironment (TME) and limiting potential side effects due to cross-reactivity against normal tissues, are also important considerations.

Cancer vaccine platforms can be divided into four major classes, as described in Table 1. Traditional vaccine platforms are frequently not optimal for the challenges of immunotherapy, which can include complex manufacturing that results in slow turnaround and higher production costs.^16^ Furthermore, most non-live vaccine platforms tend to generate predominantly CD4^+^-biased T cell responses in preclinical and clinical studies.^17^ The mechanisms of antigen presentation as well as the activation of Toll-like receptor (TLR) or pathogen-associated molecular pattern pathways might lead to the generation of CD4^+^-biased T cell response.^18^^,^^19^^,^^20^^,^^21^ Preferentially, antigens that are generated intracellularly, processed, and degraded by the immunoproteasome are efficiently presented on major histocompatibility complex (MHC) class I for the generation of more robust CD8^+^ T cell responses.^18^ Most antigenic proteins/peptides are injected into the tissue and get engulfed and processed in the endosome, and these are presented to T cells by antigen-presenting cells, skewing them to an MHC class II presentation. While mRNA vaccines are able to induce greater class I presentation on account of the antigen being generated intracellularly, mRNA might induce TLR and retinoic acid-inducible gene-1-like receptor activation, skewing the resulting responses to a more CD4^+^ T cell response with a potentially lower CD8^+^ T cell response.^19^^,^^20^^,^^21^ Tailoring a vaccine platform should allow for simple delivery, rapid and low-cost manufacturing, and enough space to allow the targeting of multiple tumor antigens simultaneously for increased breadth and generating strong cytotoxic CD8^+^ T cell responses.^17^^,^^22^ DNA vaccines have gained substantial attention in recent years as a potential important tool in the fight against cancer. Importantly, this immunogen platform utilizes the direct injection of plasmid DNA encoding a chosen tumor antigen(s) or antigen string. The DNA is taken up by host cells locally, which express the encoded antigen(s) for some period of time. These antigens are taken up by local antigen-presenting cells, which present antigen to lymphocytes.^22^ This platform is documented to stimulate both humoral and cellular immune responses including CD4^+^ and CD8^+^ T cell responses against the delivered tumor antigens.^17^ This offers important advantages for cancer immunotherapy, including the ability to generate a robust and long-lasting immune response with relatively low production costs and the flexibility to adapt to different types of tumors. It also allows for simultaneous targeting of multiple antigens to overcome antigenic heterogeneity and for the delivery of gene-encoded adjuvants to further enhance immune responses.^23^^,^^24^^,^^25^^,^^26^ Recent advances in vaccine design and delivery system enhancements such as the use of electroporation (EP) devices, gene gun, gene jet, intradermal (ID) patches, ID tattoos, and new formulations for delivery continue to enhance their therapeutic utility, improving the efficacy and providing several options for tailoring DNA-based immunotherapies for different therapeutic targets.^27^^,^^28^^,^^29^

**Table 1:** Vaccine platforms for cancer immunotherapy

Platform	Examples	Reference
Cell vaccine	Dendritic cells, whole-tumor lysates	Wculek et al.^5^; Yamano et al.^6^
Subunit/peptide vaccine	Muc1, synthetic long peptides for neoantigen	Ott et al.^7^; Stergiou et al.^8^
Viral vaccine	Adenovirus, vaccinia virus, avian pox virus, oncolytic virus	Pol et al.^9^; DiPaola et al.^10^; Majhen et al.^11^
Nucleic acid vaccine	DNA, mRNA	Zhu et al.^12^; Duperret et al.^13^; Sittplangkoon et al.^14^; Chen et al.^15^

There is a growing body of evidence supporting the potential of DNA therapeutics for cancer treatment, with multiple preclinical and clinical studies demonstrating efficacy in controlling tumors and impacting disease reported. In this review, we provide a comprehensive summary of multiple recent clinical trials evaluating DNA vaccination approaches for cancer treatment. We highlight studies that tested DNA vaccinations against different antigen classes either as a single biologic delivery or in combination with different adjuvants or immune checkpoint blockade (ICB) molecules. We also highlight some recent preclinical work expanding the DNA platform for the delivery of biologics such as monoclonal antibodies (mAbs) and novel immune cell engagers for cancer immunotherapy.

## Plasmid DNA vaccines for immunotherapy of HPV-associated diseases

HPV infections are responsible for ∼4.5% of all cancers worldwide, with the majority caused by HPV-16 and HPV-18.^30^ These two serotypes are the primary culprits responsible for most chronic HPV infections; however, additional serotypes are growing in importance. Prophylactic vaccines have been highly effective in preventing HPV infections. These vaccines induce antibodies against the viral capsid glycoprotein L1, which is not required for the virus post-infection and the progression to cancer; therefore, they are not effective in clearing established virus.^31^ Due to the large scale of preexisting chronic infections and slow vaccine uptake in some global locations, HPV-associated cancers will remain a significant global health issue for the foreseeable future.^32^ Consequently, vaccine strategies capable of eliciting robust anti-HPV immunity, particularly CD8^+^ T cell responses to help clear or control viral infection, hold great promise for addressing HPV-induced cervical and head and neck malignancies among a spectrum of HPV-driven diseases. In contrast to the L1 antigen, the viral proteins E6 and E7 are consistently expressed in HPV^+^ established infections and precancers; over time, they drive progression to HPV-associated cancers by targeting and inactivating the host p53 (E6) and Rb (E7) tumor suppressor genes.^33^ The progression of HPV-induced cervical carcinogenesis involves the transformation of normal epithelial cells into cervical intraepithelial neoplasia (CIN), which is a precancerous step in the disease chain. Precancer is classified into grades 1, 2, or 3, based on the increasing likelihood of risk, and advancing to cervical cancer.^34^ While HPV has a major role in driving cervical cancer, it is also responsible for contributing to anal, penile, vulvar, and vaginal cancers and is an important cause of head and neck cancer.^35^^,^^36^ In addition, some serotypes such as HPV-6 and -11, are major contributors to the induction of genital warts and aggressive, more rare recurrent lesions that are serious and debilitating when they are localized in the esophagus, such as recurrent respiratory papillomatosis (RRP).^37^^,^^38^^,^^39^^,^^40^ Thus, impactful therapeutic vaccines against HPV disease-causing subtypes would be potentially useful for treating diverse precancers and cancers. HPV E6 and E7 proteins from tumor-causing subtypes have become an important immunotherapeutic target for cancer vaccinations. Studies testing impact have been conducted across many platforms, including the DNA platform. Some of these studies have shown impacts in limiting or regressing HPV- associated cancer or precancers and are summarized below.

In 2015, Trimble et al*.* studied the impact of a DNA vaccine containing two DNA plasmids encoding optimized synthetic consensus E6 and E7 genes of HPV-16 and HPV-18, followed by adaptive EP by Cellectra, for inducing T cell immunity for controlling or eliminating HPV-16- and -18-associated CIN (this study was registered at ClinicalTrials.gov: NCT01304524).^41^ In a randomized, double-blind, placebo-controlled phase 2b trial, the authors reported that nearly 50% of patients receiving the DNA vaccine VGX-3100 had evidence of histopathological regression as compared to 30% of placebo patients. Viral clearance was observed in 52.3% of VGX-3100-treated participants as opposed to 25.7% of placebo participants.^41^ This important study outcome resulted in a second application in the HPV space. The subsequent study assessed VGX-3100 in both men and women for histologically confirmed anal or anal/perianal high-grade squamous intraepithelial lesion (HSIL) associated with HPV-16 and/or HPV-18 (this study was registered at ClinicalTrials.gov: NCT03499795). As in the study by Trimble et al., 50% of subjects in this trial demonstrated resolution or regression of their HSIL. This was associated with a 4.491-fold mean increase from baseline in the frequency of HPV-16/18 E6/E7-specific CD8^+^/CD137^+^/perforin^+^ peripheral blood mononuclear cells (PBMCs) at week 15 post-vaccination.^42^ Additional studies in these applications are being advanced through other collaborations. In a trial sponsored by the AIDS Malignancy Consortium, this HPV therapeutic candidate is being evaluated in the context of anal HSIL in HIV patients (this study was registered at ClinicalTrials.gov: NCT03603808), the results of which will be informative.^43^

There has been significant work in this space with additional products. Specifically, in a phase 2 randomized, open-label study of 72 patients, Choi et al. reported that candidate GX-188E, a DNA vaccine expressing HPV E6 and E7 proteins and delivered by intramuscular (IM) injection and EP, induced regression of CIN grade 3 neoplasia (this study was registered at ClinicalTrials.gov: NCT02139267). In this study more than 60% of patients had histopathological evidence of regression, and more than 70% of patients with evident histopathological regression experienced complete HPV clearance within 36 weeks.^44^ Participants with HPV clearance at 36 weeks exhibited a significant increase in interferon-γ (IFN-γ) ELISpot response to HPV E6/E7 compared to the group without histopathologic regression, highlighting a potential T cell-mediated viral clearance mechanism.

In another phase 2 trial, the safety of a uniquely designed DNA vaccine, PVX-2, where patients were boosted with tissue antigen (TA)-CIN, was tested in 12 women for HPV-16-associated low-grade squamous intraepithelial lesion/CIN1 over 12 months (this study was registered at ClinicalTrials.gov: NCT03911076). The plasmid, pNGVL4a-Sig/E7(detox)/HSP70, encoded for an oncogene, the inactivated HPV-16 E7, *Mycobacterium tuberculosis* heat shock protein 70 (HSP70), and signal peptide (Sig). Three doses were given per subject at 4-week intervals, followed by an injection of TA-CIN, a single fusion protein of HPV-16 L2, E7, and E6 proteins at week 8. All injections were well tolerated, with no adverse safety events or dose-limiting toxicities (DLTs). By month 6, 45% of study participants were HPV-16^−^, and by month 12, 64% were HPV-16^−^. Of the seven patients who experienced HPV-16 clearance, three also reverted to normal cytology. It is unknown whether the other four patients had co-infections with other HPV types, which the authors note is a limitation of the study. However, PVX-2 appeared to provide an impact in preventing HPV-16-associated lesion progression,^45^ although larger numbers of study participants and the inclusion of a placebo-controlled group would have further supported overall study interpretation.

To study whether gene-encoded adjuvants can boost relevant immune responses to these HPV vaccinations, a DNA vaccine (delivered IM with adaptive EP) against HPV-16/HPV-18 E6 and E7 proteins in combination with DNA-encoded interleukin-12 (IL-12) was tested in a phase 1 trial of newly diagnosed cervical cancer patients who had undergone chemoradiation therapy (this study was registered at ClinicalTrials.gov: NCT02172911). The vaccine demonstrated an excellent safety profile, with only grade 1 adverse events (AEs) reported. Overall, 80% of the patients generated immune responses (either cellular or humoral). Seven out of eight patients evaluable for clinical response had a complete response (CR) and one had a partial response (PR), suggesting that patients with newly diagnosed disease have a better chance to respond compared to those with metastatic/recurrent disease. The authors also observed increased programmed cell death ligand 1 (PD-L1) expression in the tumors of these patients, suggesting the combination of this therapy with anti-PD1/PD-L1 therapy might further impact response rates.^46^

To test this, Morris et al. tested DNA vaccines encoding modified E6 and E7 proteins from HPV-16/HPV-18 and DNA-delivered IL-12 as an adjuvant in combination with anti-PD-L1 durvalumab in patients with recurrent/metastatic cervical, anal, penile, vaginal, or vulvar cancers (this study was registered at ClinicalTrials.gov: NCT03439085). The vaccine and IL-12 were administered IM, and adaptive EP with the Cellectradevice was used to enhance delivery and improve immune responses. The treatment was well tolerated, with only 14% of the patients exhibiting grade 3 AEs, which did not lead to treatment discontinuation and is a previously observed side effect of anti-PD-L1 therapy. The overall response rate (ORR) reported for this study was 21% and the disease control rate (DCR) was 42%, including one patient with CR. These responses exhibited a median progression-free survival (PFS) of 3.7 months.^47^ In a larger study, the same treatment regimen was tried in a phase 1b/2a trial of HPV-associated metastatic/recurrent head and neck squamous cell carcinoma (HNSCC; this study was registered at ClinicalTrials.gov: NCT03162224). The treatment was mostly well tolerated; however, grade 2/3 treatment-related AEs (TRAEs) leading to treatment discontinuation/interruption were observed in 3 of 35 patients. The study had an ORR of 27.6%, including 4 patients with CR and a DCR at 16 months of 44.8%. There was a higher ORR in patients whose tumor cells had >25% PD-L1 compared to those whose tumor cells had <25% PD-L1 expression, suggesting patients with increased PD-L1 expression would benefit more from this therapy. The vaccine generated T cell responses against both HPV-16/HPV-18 E6 and E7 proteins in most patients, and there was a trend toward responses being correlated with improved T cell infiltration into the tumors post-therapy, suggesting a potential mechanism of action.^48^

Directing a vaccine to lymphoid tissue or to antigen-presenting cells could improve the magnitude and quality of the immune response.^49^ Hillemanns et al. tested this hypothesis with VB10.16 (coupling mutation-inactivated E6 and E7 proteins to chemokine ligand 3-like 1 [CCL3L1], an antigen-presenting cell-targeting chemokine) delivered IM in a phase 1/2a trial to evaluate safety and immunogenicity in patients with CIN2/3 lesions (this study was registered at ClinicalTrials.gov: NCT02529930). The authors observed mainly low-grade AEs, with 9% of the patients experiencing grade 3 AEs. They reported HPV-specific T cell responses in 92% of patients, reduction in lesion size in >90% of patients, and lesion clearance in 47% of the patients. The authors also observed a correlation of HPV-specific T cell responses with lesion size reduction, highlighting the important role of T cell immunity in HPV clearance and further support of the ability of the DNA platform to engender robust T cell responses in human patients.^50^ This study also highlights the potential benefit of directly targeting the antigen to an antigen-presenting cell using a cytokine *in vivo*. This approach could be interesting to test with other tumor antigens.

Finally, in an open-label randomized phase 2 trial for patients with HPV-16- or HPV-18-associated HNSCC, participants were given two or three of the following: ICB (pembrolizumab), long-acting recombinant IL-7 (GX-I7), and/or the DNA candidate GX-188E (this study was registered at ClinicalTrials.gov: NCT05286060). Preliminary findings show that the triple therapy resulted in major pathologic response in over 60% of patients. Pathologic CR was observed in over 30% of patients in this study. These data further support that this combination therapy has a manageable safety profile and promising activity among patients with surgically resectable HPV-associated HNSCC.^51^

## DNA vaccines for RRP

RRP is a rare disease, driven also by HPV infection, affecting roughly 2 in 100,000 adults in the United States.^52^ Most RRP lesions are considered benign, but some can undergo malignant transformation. Additionally, RRP patients are at an increased risk of developing laryngeal neoplasias and carcinomas.^53^ Of concern, RRP patients face repeated throat surgeries for removal of the benign lesions that can grow continuously and thus face increased morbidities resulting from changes in voice production, difficulty in breathing, and adverse effects of repeated anesthesia, as well as economic challenges.^39^^,^^54^^,^^55^ The E6 and E7 proteins of HPV-6 and HPV-11 primarily, and HPV-16 and HPV-18 more secondarily, are implicated in the pathogenesis of RRP, and targeted immunotherapy against these proteins could provide significant benefits to RRP patients for improving their quality of life.^52^^,^^56^

In a small, two-patient study, Aggarwal et al., tested a DNA vaccine (IM + adaptive EP) against E6 and E7 proteins of HPV-16/-18 with gene-coded IL-12 in RRP patients (this study was registered at ClinicalTrials.gov: NCT02241369). The therapy was well tolerated, and the vaccine generated strong antibody responses lasting up to 21 weeks post-last dose of the vaccine in both patients and induced strong CD8^+^ T cell responses evidenced by increased CD8^+^ T cell activation upon stimulation with antigenic peptides *ex vivo*. The vaccine also reduced the need for surgical intervention as one patient did not require any surgery for over 900 days post-vaccination and another one experienced an over 3-fold decrease in the need for surgery, going from surgery every 180 days on average to over 500 days without surgery.^52^

Building upon this initial data, Morrow et al. tested a DNA vaccine (IM + adaptive EP) targeting the E6 and E7 proteins of HPV-6 and HPV-11 along with gene encoded IL-12 for RRP immunotherapy in a phase 1/2 trial of 32 patients (this study was registered at ClinicalTrials.gov: NCT04398433). The therapy was well tolerated with either grade 1 or 2 AEs observed in any treatment-related event. Overall, 100% of patients demonstrated a peripheral immune response measured either by IFN-γ ELISpot, intracellular cytokine staining, or T cell receptor-β (TCR-β) sequencing. A total of 81.3% patients experienced a decrease in the number of surgical interventions in the year following therapy initiation, including 28.1% of the patients who required no surgeries (CR) and 43.8% of the patients who had a reduction in the number of surgeries by 50%–99% (PR).^57^

Overall, these studies demonstrate exciting results in targeting HPV subtypes 6, 11, 16, and 18, with strong antigen-specific T cell responses generated in the majority of treated patients. Highly promising is that the treatment would often lead to the complete clearance of HPV viral burden as well as tumors. The use of IL-12 as a gene-encoded adjuvant improved immune responses and demonstrates that newly diagnosed patients could derive significantly more benefits compared to those with recurrent or metastatic disease. Additional efficacy studies in HPV-associated cervical and head and neck cancer patients are important to extend these potential products toward licensure. The addition of anti-PD-1/PD-L1 could further boost immune responses, especially in patients whose tumors express high levels of PD-L1. The combination DNA vaccine described above showed benefit for RRP patients, who often require multiple surgeries every year. The benefits derived from the vaccine would result in fewer annual surgeries and related hospital visits, improving the quality of life and significantly easing the economic burden on the healthcare system. The study team reported that the data generated in the described study of RRP therapy may support biologics license application, with an additional confirmatory study to be developed at the same time as submission. The DNA vaccine platform can accommodate multiple antigens; accordingly it can be studied incorporating additional potentially important HPV T cell targets that are implicated in oncogenesis.

## DNA vaccines with adjuvants

Adjuvants enhance immune responses to vaccines, potentially reducing the required dose or number of doses and allowing for more consistent responses in diverse populations, including older patients.^58^ They can also be tailored to promote specific, more targeted immune responses, such as humoral or cellular immunity.^59^^,^^60^ Cytokines including IL-2, IL-7, IL-15, IL-21, and IL-33 have been reported to boost antigen-specific CD4^+^ and CD8^+^ T cell responses, many in gene-delivered forms.^61^^,^^62^^,^^63^^,^^64^^,^^65^ In the case of DNA vaccination specifically, several different adjuvants such as IL-15, IL-12, CCL27, and adenosine deaminase have been studied for their potential to boost cellular and humoral responses or redirect them to specific anatomical sites.^66^^,^^67^^,^^68^^,^^69^ Additionally, IL-21 and IL-15 promote the formation of long-term memory T cell responses, which can be critical in preventing or delaying tumor recurrence.^70^ Chemokine adjuvants have also been described as useful for improving immune responses and redirecting immune responses to different anatomical sites. Several chemokines such as CCL3 and CCL20, C-X-C motif chemokine ligand 10, CCL21, granulocyte-macrophage colony-stimulating factor (GM-CSF), and CCL5 have been shown to enhance the efficacy of anti-tumor vaccines via distinct mechanisms in mouse models of different cancers.^71^^,^^72^^,^^73^^,^^74^^,^^75^^,^^76^ Several of these approaches have been advanced into clinical studies and are reviewed below.

## DNA vaccines for prostate cancer immunotherapy

Currently, prostate cancer is the only cancer type with a licensed therapeutic vaccine (Provenge).^77^ Provenge is an immune cell therapy that was developed to drive immune responses against prostate cancer and slow disease progression. The limitations of this therapy are that it is very difficult to produce and only extends the patient’s lifespan less than 6 months. Improving outcomes in this space is important. Despite several advances in therapies, prostate cancer remains the fifth-leading cause of cancer deaths globally.^1^ While prostate cancer has a low mutagenic burden, it benefits from the expression of several TAAs such as prostatic acid phosphatase (PAP), prostate-specific membrane antigen (PSMA), PSCA, PCTA, and STEAP1, all of which could be appropriate targets for therapeutic vaccines.^78^^,^^79^

In a phase 2 clinical trial, McNeel et al. compared a DNA vaccine (delivered ID, no EP) targeting PAP adjuvanted with GM-CSF to GM-CSF therapy only in patients with recurrent, hormone-sensitive prostate cancer (this study was registered at ClinicalTrials.gov: NCT01341652). In this trial, the investigators did not observe any difference in metastasis-free survival (MFS) between the two groups. This was likely because the vaccine only produced short-term anti-PAP T cell responses, which were rapidly lost.^80^ To boost immune responses, the same group performed another phase 2 clinical trial combining DNA vaccine against PAP in combination with the anti-PD-1 antibody nivolumab in castration-sensitive non-metastatic prostate cancer patients (this study was registered at ClinicalTrials.gov: NCT03600350). If prostate-specific antigen (PSA) levels were higher at 4 weeks compared to baseline, they further adjuvanted the therapy regimen with GM-CSF. A total of 4 out of 19 (21%) patients had a PSA decline of >50%, and they observed a significantly enhanced PSA doubling time of 25.6 months while patients were on therapy, compared to a PSA doubling time of 5.9 months prior to commencement of therapy. The authors did report one patient with a grade 4 AE and two other patients with grade 3 AEs. Immunological analysis demonstrated 15 out of 19 patients developed antigen-specific immune responses. In 42% of the patients, these responses were observed at two time points, indicating longer-lasting immune responses over their previous trial.^81^

Building on this work, the team recently reported clinical data from long-term follow-up of an anti-PAP DNA vaccine with GM-CSF in non-metastatic castration-sensitive prostate cancer (nmCSPC) (this study was registered at ClinicalTrials.gov: NCT00582140) and non-metastatic castration-resistant prostate cancer (nmCRPC) (this study was registered at ClinicalTrials.gov: NCT00849121). For the nmCSPC patients, at 15-year follow up, 5 of 22 patients were alive, 8 of 22 died of prostate cancer, and 9 of 22 died of other causes. Sixteen patients developed metastasis, with a median time to develop metastasis of 8.2 years. For nmCRPC patients, 8 of 17 patients were alive at the 5-year follow-up time point. The median time to metastasis development was 1.4 years. The authors observed long-lasting (several years) anti-PAP immune responses in 60% patients in both trials.^82^ The time to development of metastasis is similar to the current MFS time for nmCRPC patients,^83^ suggesting minimal if any clinical benefit of this therapy. Given the safety and tolerability of this regimen, the treatment might still provide meaningful improvement in the quality of life of these patients. These trials, while not statistically significant, suggest the potential benefits of using DNA vaccines against PAP with GM-CSF as an adjuvant.

In another study of mCSPC patients, Kyriakopoulos et al. studied DNA vaccine targeting the androgen receptor ligand binding domain (AR LBD) adjuvanted with or without GM-CSF. The study reported no grade 3 or higher AEs. A total of 16 in 30 patients generated AR LBD-specific T cell responses that were higher than baseline. The authors reported some evidence of antigen spreading as they also observed PAP-specific T cell responses in 9 of 30 patients, despite PAP not being included in the vaccine. Overall, 27/40 patients were progression-free at 18 months. While they observed no significant differences in immune responses or time to progression in groups with or without GM-CSF, they did report a potential benefit in time to castration resistance and PSA rise in patients who developed anti-AR LBD T cell responses.^84^ Follow-up studies would be important to confirm and extend these data.

In a phase 1/2 study, Shore et al. studied the delivery of DNA vaccine (delivered IM with adaptive EP) against PSA and PSMA with gene-encoded IL-12 also being delivered as a cytokine adjuvant (this study was registered at ClinicalTrials.gov: NCT02514213). The study evaluated 62 prostate cancer patients who had undergone surgery/radiation therapy but had rising PSA levels. Shore et al. found that 11.3% of patients had grade 3 TRAEs, although none of them led to withdrawal from the study. Approximately 25% of the patients developed antibodies against PSA or PSMA, with PSA driving higher seroconversion and the authors reporting slightly better seroconversion rates in groups that co-received IL-12. The vaccine also elicited T cell responses against both PSA and PSMA, although the benefit of IL-12 was limited for T cell immunity. The authors did observe higher expression of PD-1 on antigen-specific T cells, suggesting that these patients could benefit from the addition of anti-PD-1 to the treatment regimen. The vaccine also slowed the rise in PSA levels compared to when patients received the initial dose of therapy, and it significantly slowed PSA doubling times, highlighting the benefit of vaccine therapy in this patient population.^85^

These studies highlight the potential advances of vaccine therapy for prostate cancer patients. Although most patients generated humoral and cellular responses to the limited prostate cancer antigens studied, which appeared to be boosted by GM-CSF and IL-12 adjuvants, the clinical responses were limited. This could be due to transforming growth factor-β (TGF-β)-mediated immunosuppression, which is a major challenge for prostate cancer immunotherapy.^79^ Combination therapies that can overcome TGF-β-mediated immunosuppression or with anti-PD-1 therapies to further boost T cell activity could yield improved responses in the clinic. Focusing on more immunogenic formulations and potentially additional vaccine combinations may also be important.

## DNA vaccines for breast cancer immunotherapy

Breast cancer is one of the most commonly diagnosed cancers globally and was the cause of almost 700,000 deaths in 2020.^86^ While traditionally considered a cold tumor, recent data have shown that the presence of tumor-infiltrating lymphocytes correlates with improved prognosis and response to chemotherapy in breast cancer patients. Additionally, immune gene signatures are associated with improved patient prognosis, especially in patients with human epidermal growth factor receptor 2^+^ (HER2^+^) breast cancer and triple negative breast cancer (TNBC), the two most aggressive types of cancer.^87^ This highlights that breast cancer can benefit from immunotherapies, and newer strategies that can better exploit this are needed.

In a phase 1 study of advanced-stage ERBB2^+^ breast cancer patients, Disis et al. ID (without EP) vaccinated patients with constructs targeting the intracellular domain (ICD) of ERBB2, studied at three different doses: 10, 100, and 500 μg (this study was registered at ClinicalTrials.gov: NCT00436254). All patients received a tetanus toxoid vaccination as a potential but unusual adjuvant approach prior to the first dose of the vaccine and GM-CSF delivery with every dose of the vaccine. Overall, the treatment was well tolerated, with the majority of AEs being grade 1 or grade 2, with a single grade 3 AE observed among the 66 patients treated. Using a 10-day IFN-γ ELISpot assay, the authors observed an increase in T cell responses against ERBB2^+^ ICD in a dose-dependent fashion, lasting up to 60 weeks in patients who received the two higher doses of the vaccine. Interestingly, they observed an inverse relationship between long-term T cell immune responses and DNA persistence (measured by PCR assay on DNA extracted from a skin biopsy of the injection site) at the injection site. They observed evidence of DNA persistence in patients who received the 500-μg dose, which was associated with a decrease in T cell immune responses once patients stopped receiving vaccinations. The authors did describe a minor improvement in overall survival (OS) and PFS in patients who received the 100-μg vaccine compared to the two other doses, although these differences were not statistically significant.^88^ The median OS (mOS) was not reached in any of the groups, which is an improvement in mOS of patients receiving HER2 targeting mAb therapy alone (5-year OS of 54%).^89^ The study was unusual in that vaccine responses increased after the immunizations were completed. Additional investigation of this study to understand the parameters described and to consider next steps for immune enhancement and further trial advancement may be important.

In a phase 1 dose-escalation study of STEMVAC, the authors tested a DNA plasmid (administered ID) targeting the CD105/YB-1/SOX2/CDH3/MDM2 multi-epitope adjuvanted with recombinant human GM-CSF (rhuGM-CSF) for patients with advanced HER2^−^ breast cancer (this study was registered at ClinicalTrials.gov: NCT02157051). The patients received either 150, 300, or 600 μg of the DNA vaccine. The authors observed 1 antigen-specific IFN-γ-secreting T cell per 2,500 PBMCs at week 16, with a maximal response of 1:1,500 by week 60 in patients receiving the middle dose. Increasing dose did not significantly improve this response, but boosting immunizations at the 300-μg dose increased the incidence and breadth of IFN-γ secretion as measured by the number of antigens against which immune responses were observed. Additionally, at 16 weeks no increase in antigen-specific IL-10-secreting T cells, regulatory T cells (Tregs), or myeloid-derived suppressor cells (MDSCs) at any dose were observed.^90^ For testing the efficacy of STEMVAC, two subsequent phase 2 trials are currently enrolling for TNBC (NCT05455658) and stage IV non-small cell lung cancer (this study was registered at ClinicalTrials.gov: NCT05242965). It will be important to build on the early immune data to, hopefully, translate into statistically significant impacts on patient outcomes in these later trials, which were not reported in the early studies.

In another phase 1 trial, Stanton et al. studied immunization with DNA immunogens encoding three antigens: HER2, insulin-like growth factor binding protein-2 (IGFBP-2) and insulin-like growth factor 1 receptor with rhuGM-CSF adjuvant in non-metastatic HER2^−^ breast cancer patients in remission with no evidence of disease (this study was registered at ClinicalTrials.gov: NCT02780401). Patients received three doses of the treatment ID and were grouped into three different doses of 150, 300, or 600 μg per dose. The intermediate dose of 300 μg led to the strongest and longest-lasting T cell responses compared to the other doses. There were no significant safety concerns and no antigen-specific MDSCs, Tregs, or T helper 2 responses were observed.^91^ Detailed immunology responses and clinical outcomes remain to be seen. No statistically significant impact on patient outcomes has been reported.

These early-stage clinical trials implicate the ability of GM-CSF to potentially enhance immune responses. One issue is that most of these studies used lower doses than were delivered in the earlier-described HPV immunizations. More consideration of this difference may be helpful in future studies. More detailed characterization of these responses will be highly informative. Understanding the lack of dose escalation and the immune responses observed would be important. Building on these trials to study detailed immunology responses and controlled trials will provide important information about next steps in the development of these immunotherapeutic tools for breast cancer.

## DNA vaccines for neuroblastoma

Neuroblastoma is a cancer of the sympathetic nervous system and is responsible for roughly 10% of pediatric cancer deaths.^92^^,^^93^ Neuroblastoma is among the least-mutated tumors, resulting in it being classified as an immunologically cold tumor. Despite this, the presence of tumor-infiltrating lymphocytes is associated with improved prognosis in high-risk neuroblastoma patients, suggesting that vaccines that elicit strong CD8^+^ T cells and have them home to the tumor could potentially benefit neuroblastoma patients.^94^

A novel adjuvanted DNA antigen cocktail encoding multiple tumor antigens was developed and studied in a neuroblastoma phase 1 clinical trial (this study was registered at ClinicalTrials.gov: NCT04049864). Proleskovskaya et al. treated six neuroblastoma patients with DNA vaccine encoding the antigens for tyrosine hydroxylase phox2b, Preferentially Expressed Antigen of Melanoma, and survivin along with lenalidomide as an adjuvant for immune stimulation. The vaccine was delivered rather differently, in an attenuated strain of salmonella, to serve as a plasmid transfer vector and to further adjuvant the vaccine. The vaccine was well tolerated and five of six patients generated antigen-specific T cell responses. While the vaccination therapy suggested the potential to improve event-free survival in this small group of patients, other data for impact on survival and the longevity of the induced immune responses were not available.^95^ Clinical outcomes in larger studies with impact on tumor regression would be important for next steps with this vaccine.

Overall, the studies above support that different deliveries and types of DNA immunizations can elicit cellular and humoral responses against diverse cancer types. GM-CSF increased the frequency of T cell responses, and IL-12 boosted antibody responses at a low level in prostate cancer patients.^82^^,^^85^ The DNA immunizations combined with adjuvants demonstrate some increase in T cell frequency, even in immunological cold tumors such as breast cancer and neuroblastoma. However, the studies did not show significant differences in OS, which will require larger studies to address.

## DNA immunization with ICB

**Figure 3:**
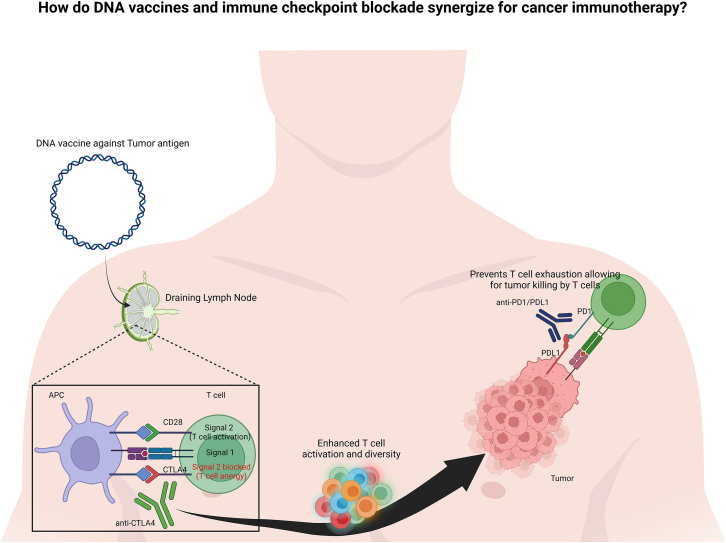
Mechanism of action for how anti-CTLA4 and anti-PD-1/PD-L1 antibodies synergize with DNA vaccines for cancer immunotherapy

ICB uses antibodies to block key immune receptors, like cytotoxic T-lymphocyte associated protein 4 (CTLA4) and PD-1, that normally limit T cell activity or cause T cell exhaustion. The antibodies function by blocking these receptors from interacting with their respective ligands, which allows for improved immune priming and reduced T cell exhaustion. In the decade following the first anti-CTLA4, ipilimumab’s US Food and Drug Administration approval in 2011, ICB has transitioned to become the first-line therapy against many unresectable tumors.^96^^,^^97^ Anti-CTLA4 therapy works by direct depletion of immunosuppressive Tregs as well as blocking the interaction of CTLA4 with CD80/CD86, removing the negative CTLA4-CD28 interaction. This permits improved binding of CD28, a co-stimulation molecule, with CD80/CD86 enhancing T cell activation, resulting in a greater diversity of T cell clones induced, which can target tumor antigens.^98^^,^^99^ Anti-PD-1/PD-L1 therapy works by blocking the interaction of PD-1 expressed on T cells with its ligand PD-L1, which is expressed on tumor cells or immunosuppressive cells such as MDSCs and Tregs. This prevents the inhibition of T cell activity in the TME, allowing T cell-mediated cytotoxicity of tumor cells.^100^ Anti-PD-1/PD-L1 therapy can also function in the tumor-draining lymph nodes and drive enhanced T cell co-stimulation, which allows for better activation of cytotoxic T cells that can then traffic to the tumor site and kill tumor cells.^100^^,^^101^ ICB therapy faces numerous challenges, including resistance-driven resurgence, wherein tumors initially respond to therapy but eventually regrow due to immune evasion, upregulation of alternate immune checkpoint molecules, T cell dysfunction, and the presence of immunosuppressive factors in the TME.^102^ Toxicity, resulting in immune-related AEs (irAEs), is another challenge with the broader applications of ICB therapy.^103^^,^^104^ Combination therapies pose the best defense to minimize resistance and maximize duration of response. However, dual ICB therapy poses an increased risk of high-grade irAEs.^105^ DNA vaccines coding for tumor antigens can synergize with different types of ICBs and can significantly enhance the efficacy of ICB therapy (Figure 3). Accordingly, preclinical studies combining DNA vaccines with ICB show great potential.^106^^,^^107^^,^^108^ DNA vaccines in combination with ICB may also be very useful in expanding the number of TAAs targeted and the diversity of immune response generated, which can further improve response durability and minimize DLTs.^109^

## DNA vaccines for prostate cancer immunotherapy with ICB

In a randomized phase 2 trial, the efficacy of pTVG-HP, a DNA plasmid encoding PAP, adjuvanted by GM-CSF in combination with the anti-PD-1 antibody pembrolizumab, was studied in 66 patients with mCRPC (this study was registered at ClinicalTrials.gov: NCT02499835). The authors report results from 40 patients on whom they tested two different dosing schedules: (1) vaccination and pembrolizumab was given every 3 weeks or (2) vaccination was given every 2 weeks and pembrolizumab was given every 4 weeks. In the first group, 4 in 20 patients had grade 3/4 AEs, including one that led to discontinuation. In the second group, 13 in 20 had grade 3/4 AEs, none of which led to discontinuation. Two patients in the second group had grade 2 irAEs, which were attributed to pembrolizumab and led to stopping pembrolizumab treatment while continuing to receive the vaccine. A total of 10% of the patients in the first group and 30% of the patients in the second group developed PAP-specific CD4^+^ and CD8^+^ T cells, which were detectable for up to 1 year. Across both groups, 35% of the patients exhibited any level of PSA decline, including 4 of 40 who experienced a greater than 50% decline in PSA levels and 3 in 40 who demonstrated a greater than 90% decline in PSA levels. Surprisingly, there was only a weak correlation between decline in PSA levels and anti-PAP immune responses, and only one patient who had a greater than 50% decline in PSA level had any detectable anti-PAP T cell response. Out of 16 patients who had radiologically measurable disease prior to therapy initiation, 5 had evidence of tumor volume decrease, including 1 that was a confirmed partial responder. The mOS for the trial was 22.9 months.^110^ This figure is better than the historical mOS for this patient population on anti-PD-1 therapy alone (∼9.5 months).^111^ The authors identified the need to vaccinate against additional prostate cancer antigens to further boost immune responses. Strategies to overcome the TGF-β-mediated immunosuppression could also help immunotherapy for prostate cancer patients. More work in this area is needed.

## DNA vaccines for glioblastoma with ICB

Glioblastoma multiforme (GBM) is the most common and most aggressive form of brain cancer. While traditional treatment modalities like surgery followed by radiation and chemotherapy are effective temporarily, the tumors rapidly recur, leading to an mOS rate of 14–17 months and a dismal 5-year survival rate of 5%.^112^ GBM tumors can express a wide range of targets such as Tert, WT1, PSMA, EGFRvIII (endothelial growth factor receptor vIII), IL13Rα2, and HER2, requiring complex formulations and multiple targets for consideration in immunotherapeutic vaccination.^112^^,^^113^^,^^114^^,^^115^ Additionally, GBM immunotherapy remains challenging due to the presence of the blood-brain barrier, which often prevents the flow of therapies to the site of the tumor. An immunosuppressive microenvironment further increases challenges for GBM immunotherapy.^116^

In a phase 1/2 trial for newly diagnosed glioblastoma patients, Reardon et al. tested the delivery of multiple plasmid-encoded antigens, all designed as microconsensus antigens to help break central immune tolerance.^113^ The multiantigen cocktail consisted of three antigens—hTert, WT1, and PSMA—and they were co-formulated for direct IM injection using Cellectra adaptive EP delivery. Patients also received plasmid-encoded IL-12 as a cytokine adjuvant as part of the vaccine cocktail, and they were treated with an anti-PD-1 antibody, cepilimumab, to further support the T cell responses induced by the vaccine (this study was registered at ClinicalTrials.gov: NCT03491683). The study had two cohorts corresponding to rapid progressors and slower progressors associated with their *O*-6-methylguanine-DNA methyltransferase (MGMT) status: cohort A, MGMT-unmethylated patients, and cohort B, MGMT-methylated patients. The unmethyated patients can repair DNA breaks induced by chemotherapy better than the MGMT-methylated patients, so they tend to progress significantly quicker.^117^ The therapy was well tolerated, with most AEs being ≤grade 2 in this trial of 52 patients. The authors observed antigen-specific CD4^+^ and CD8^+^ T cell responses, with CD8^+^ T cell responses demonstrating the expression of lytic molecules perforin and granzyme A. The mOS for cohorts A and B was 17.9 months and 32.5 months, respectively. The study revealed a 23% and 28% reduced risk of death in cohorts A and B, respectively. They also discovered a correlation between pretreatment gene expression signature and OS at 18 months in the MGMT-unmethylated patient population.^113^ The mOS in this initial trial was a small improvement for these patients compared to anti-PD-1 therapy alone (13.4 months and 28.9 months for newly diagnosed MGMT-unmethylated and MGMT-methylated patients, respectively).^118^ This study showed a consistent immunology signature and represents a promising therapeutic option for additional study for this difficult-to-treat disease.

In an open-label phase 1/2 study, Wick et al. examined the response to the tumor overexpressed antigen vascular EGFR2 encoding DNA plasmid VXM01 with the anti-PD-L1 antibody avelumab in patients with progressive glioblastoma (this study was registered at ClinicalTrials.gov: NCT03750071). Over 96 weeks, VXM01 was given orally in a bacterial Ty21 as carrier for the plasmids four times weekly, and avelumab was given intravenously (IV) twice weekly. During the 2-year observation period, no treatment-related toxicities were observed, and three PRs were reported with two patients reaching 12 months without disease progression. They noted an increase in intratumoral CD8^+^ T cells; however, with ORR at just 12%, the authors indicate that improvement in impact will be important and suggest that future studies be conducted with patient selection based on using immune biomarkers to preselect potential best responders.^119^

Overall, these initial trials highlight that DNA vaccines in combination with ICB can be delivered safely with high tolerability. Some formulations and deliveries are promising to potentially impact GBM tumors, where typically immunotherapies have not been very effective. More work targeting this disease is important as new, more effective approaches are a major goal. The pretreatment gene expression signature identified by Reardon et al.^113^ can be used as a screen to select patients who are most likely to respond to the vaccine therapy, and further tinkering with the vaccine antigens and regime could be of significant value in additional clinical studies.

## DNA vaccines for skin cancers with ICB

Skin cancers such as melanoma, Merkel cell carcinoma (MCC), and SCCs exhibit some of the highest responses to ICB therapy, including anti-PD-1 therapy. Despite these remarkable results, only about 50% of the patients benefit, and several patients relapse after an initial response.^120^ Newer therapies that can improve the response rates for anti-PD-1 therapy would significantly benefit skin cancer patients.

In a phase 1 trial for cutaneous melanomas, Markowitz et al. tested IFx-Hu2.0, a plasmid encoding part of the Emm55 M-like surface protein of *Streptococcus pyogenes* intratumorally (IT) (this study was registered at ClinicalTrials.gov: NCT03655756). No DLTs were encountered after 30 days, and the vaccine induced the activation of IFN pathways via TLRs. Furthermore, three of four patients who were previously refractory to anti-PD1 achieved benefit from retreatment with anti-PD-1 antibodies, indicating a potential for synergistic improvement of the therapy in conjunction with anti-PD-1 antibodies.^121^

A follow-up study of the IT IFx-Hu2.0 included 22 patients with checkpoint inhibitor-resistant MCC and cutaneous SCC (cSCC) (this study was registered at ClinicalTrials.gov: NCT04160065). All patients had their disease progress on anti-PD(L)-1 therapy. MCC patients who do not respond to ICB have few therapeutic options and have a mOS of 13 months, with a 2-year survival rate of 8.2%.^122^ cSCC patients who do not respond to ICB can be treated with EGFR inhibitors, and data from early-stage clinical trials or case reports suggests a median PFS of 4–25 months in these patients.^123^ In this non-randomized phase 1 trial, 56% of MCC patients and 14% of cSCC patients rechallenged with ICB led to an objective response, with durations lasting over 20 months. The IT injections were well tolerated, maintained high safety levels, and only one high-grade toxicity event was observed, possibly associated with the therapy. As such, the authors indicate that IFx-Hu2.0 induces an immune priming effect that reduces resistance to ICB, thereby leading to a more durable response and PFS.^124^

In a phase 2 open-label umbrella study in patients with unresectable melanoma, 16 patients received (IM) the DNA vaccine SCIB1 using the PharmaJet Stratis needle-free injection device system and either pembrolizumab or nivolumab with ipilimumab up to 10 times over 2 years at 8 mg per dose (this study was registered at ClinicalTrials.gov: NCT04079166). SCIB1 targets T cell epitopes from TRP-2/gp100 in an antibody framework for Fc targeting. All trial participants had stage IV disease. At the 13-week time point, 8 of 10 evaluable patients had an objective response according to RECIST 1.1 criteria. Furthermore, between 31% and 95% reduction in tumor volume was observed between 13 and 25 weeks. With no clinically meaningful AEs, further recruiting is ongoing to observe responses in additional patients.^125^ The response rates for the nivolumab + ipilimumab combination treatment in advanced melanoma patients is about 58%.^126^ If the results from the SCIB1 trial hold up in controlled trials with larger numbers, then this could represent a significant advancement in therapy for patients with advanced melanoma.

The two studies with IFx-Hu2.0 demonstrate that skin cancer patients previously refractory to anti-PD-1 therapy may benefit from anti-PD-1 therapy post-vaccination. The vaccine most likely leads to the generation of newer T cell clones against tumor antigens, which then further benefit from anti-PD-1 therapy, allowing a more effective T cell response to perhaps drive improved tumor regression. While the efficacy of this therapy needs to be established in larger trials, this modality, if reproduced, represents a potential option for treatment of skin cancers that have previously not responded to anti-PD-1 therapies. SCIB1 technology represents a novel approach that can also engage the innate immune system via Fc targeting. These represent important tools for further study for their impact in larger trials, which will be followed closely.

Overall, these studies demonstrate that DNA formulations in combination with ICB can impact GBM, providing a complementary tool for this extremely difficult-to-treat cancer. Skin cancer patients who progress on anti-PD-1 therapy have limited treatment options. The combination of DNA vaccines with anti-PD-1 can generate newer T cell clones, which can be boosted by the anti-PD-1, providing a newer therapeutic approach. It will be important to see newer controlled studies with the potential to improve OS in a larger number of patients.

## Personalized DNA vaccines

**Figure 4:**
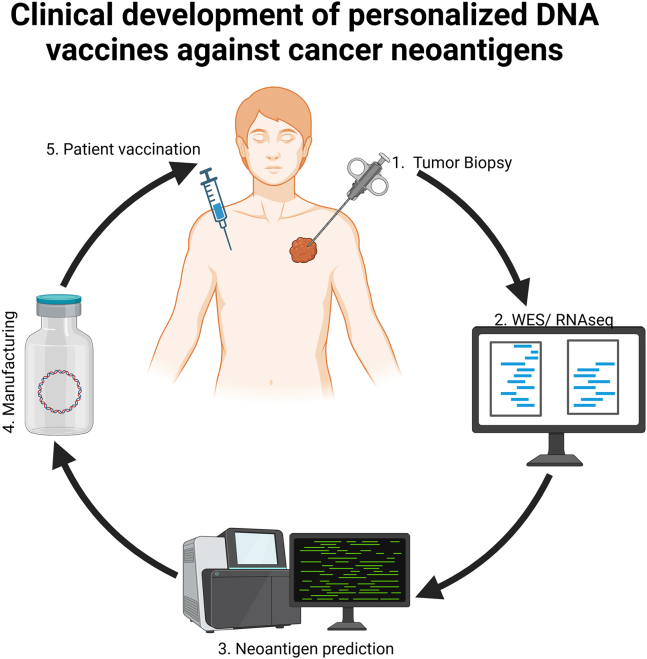
Clinical development of DNA vaccines targeting neoantigens

Among the newest approaches to cancer immunotherapy are targeting the individual genetic errors that each person’s tumor makes as disease progression occurs. Neoantigens are derived from somatic mutations in cancer cells. Tumors consist of rapidly dividing cells that have at least partially escaped immune regulation, resulting in them immunologically ignoring the tumor’s mistakes that occur during tumor cell division. It is speculated that this process may be enhanced by either inherited or *de novo* function loss occurring in enzymes that ensure the fidelity of genome replication and thus increase mutational abundance.^127^ These errors result in the expression of foreign antigenic proteins, which can be recognized by the immune system. Neoantigens represent ideal targets for therapeutic cancer vaccines because their expression is limited to tumor cells only, and the T cells that can recognize the neoantigens are not subject to central tolerance.^17^^,^^128^ Neoantigens are predominantly derived from non-synonymous mutations (mutations that lead to a change in the protein sequence^129^), and they can also be derived from insertion or deletion mutations, resulting in changes in open reading frames, as well as gene-RNA fusions.^130^^,^^131^ Because every tumor evolves differently, each patient develops a unique tumor mutation profile. This necessitates generating highly personalized vaccines for every patient. Several preclinical studies using DNA vaccines targeting neoantigens derived from different tumor types have been reported, demonstrating the generation of neoantigen-specific CD4^+^ and CD8^+^ T cell responses and, in some cases, impressive control of tumor growth in animal models.^17^^,^^108^^,^^128^^,^^132^^,^^133^^,^^134^^,^^135^ The clinical pathway to delivering neoantigen vaccines is demonstrated in Figure 4. The process usually begins with tumor biopsy to collect a tumor sample. A small piece of normal tissue surrounding the tumor that is preferred due to tissue matching or the patient’s PBMCs are collected for comparison with the tumor genome and mRNA. This is followed by whole-exome sequencing (the protein coding sequence of the open reading frame) and RNA sequencing to confirm expressed mutation and obtain the copy number of neoantigens from the tumor. Next, mutations are identified and prioritized based on several factors, including MHC class 1 binding affinity prediction via *in silico* methods,^136^^,^^137^ tumor expression levels, and differentiation from self. Once neoantigens are prioritized, they are encoded into a single vector. Different groups use different strategies to build them into a single vector. The individual neoantigens have been reported to be separated by furin, P2A, or other methods for antigen cleavage to promote neoantigen string processing.^128^^,^^138^ The P2A system is a 22-amino acid peptide that allows for the efficient cleavage of the translated product and for the expression of two or more proteins from a single open reading frame.^139^ Some groups use flexible linkers to sew the neoantigen strings together.^140^^,^^141^

Cullinan et al. conducted a phase 1b study of a DNA vaccine targeting neoantigens from pancreatic ductal adenocarcinoma (PDAC) patients who had undergone surgical resection and had no evidence of disease (this study was registered at ClinicalTrials.gov: NCT03122106).^142^ PDAC patients after surgery have a median survival of 22.1 months, with a 5-year survival rate of 17%, highlighting an urgent need for newer therapies.^143^ The authors found the vaccine to be safe and well tolerated, with no high-grade AEs. The authors targeted an average of 13 neoantigens per patient (range 5–20). Across the two patients who had received all doses of the vaccine, they observed an increase in IFN-γ ELISpots (following a 12-day *in vitro* culture) against 7 of 17 (∼41%) neoantigens tested, although the breakdown between CD4^+^ and CD8^+^ responses was not reported.^142^ The same group conducted a phase 1 clinical trial targeting neoantigens in TNBC patients who had undergone surgery and neoadjuvant chemotherapy (this study was registered at ClinicalTrials.gov: NCT02348320). In this study, they vaccinated 18 patients (average 11 neoantigens per patient, range 4–20) and observed neoantigen-specific T cell responses in 14 of 18 patients. The neoantigen vaccines predominantly elicited CD8^+^ T cell responses, although the authors also observed CD4^+^ T cell responses. While the study was not powered to study clinical responses, at a median follow-up of 36 months, they reported a recurrence-free survival rate of 87.5% in the vaccinated patients. This compares favorably with historical institutional control TNBC patients of similar stage disease treated with neoadjuvant chemotherapy who had a recurrence-free survival of 49% at 36 months. Larger randomized studies with appropriate controls are needed to further establish these results, but this study highlights the potential benefit of neoantigen vaccines in this difficult-to-treat patient population.^137^

In another study, Szymura et al. treated lymphoplasmacytic lymphoma (LPL) patients using DNA vaccine encoding the heavy and light chains of the B cell receptor, fused to chemokine CCL20 to improve targeting to the antigen-presenting cells (this study was registered at ClinicalTrials.gov: NCT01209871). The vaccine was overall well tolerated with 1 in 9 patients developing grade 3 AEs, which resolved within 2 months of symptomatic presentation. One in 9 patients developed a minor response, 4 progressed to symptomatic disease requiring additional therapy, and the remaining patients were classified as stable disease (SD) based on response criteria from the Sixth International Waldenström’s Macroglobulinemia Workshop Consensus Panel. The median time to progression was ∼2.3 years. The vaccine generated CD4^+^ and CD8^+^ T cell responses in all patients and reduced the clonal tumor subpopulation in the mature B cell compartment but not the LPL plasma cell compartment.^144^ The median time to first vaccination from initial diagnosis was 2.2 years. Advancements in sequencing technologies and vaccine manufacturing can improve this timeline and potentially enhance response rates. Additional work to improve this approach for this patient population remains important.

Given that neoantigens are the primary target of ICB-induced T cells, a combination of vaccines targeting neoantigens and ICB could have a synergistic effect. Shah et al. examined this in a phase 1 clinical trial of metastatic hormone-sensitive prostate cancer (mHSPC) patients testing a combination of DNA vaccines (delivered IM with EP) targeting PSA, personalized neoantigens, and ipilimumab/nivolumab after patients had undergone chemotherapy and androgen-deprivation therapy (this study was registered at ClinicalTrials.gov: NCT03532217). The therapy was generally well tolerated, with mainly low-grade AEs, although 2 in 15 patients did have grade 3 TRAEs. While the immunology data remain to be seen, the authors reported a 2-year OS rate of 75%.^145^ The current standard of care therapy for high-risk mHSPC patients is triple therapy with androgen deprivation, androgen receptor inhibition, and chemotherapy, with a 2-year OS rate of ∼85%.^146^ The vaccine in combination with ipilimumab/nivolumab had a better safety profile as 66.1% of the patients receiving the triple therapy had grade 3/4 AEs, resulting in 10% of the patients stopping therapy. Additional immunology and clinical response data from larger trials are potentially important.

Recently, Yarchoan et al.^136^ conducted a phase 1/2 trial of 36 hepatocellular carcinoma (HCC) patients whose tumors had progressed on multiple prior lines of treatment. This trial is one of the few to look at later-stage disease in a cold tumor. The authors treated patients with existing tumors (including several who also had metastatic lesions) with personalized neoantigen vaccine, plasmid-encoded IL-12 formulated as part of the neoantigen vaccine as cytokine adjuvant, and pembrolizumab (this study was registered at ClinicalTrials.gov: NCT04251117). The plasmids encoding vaccine and IL-2 were delivered ID with adaptive EP using the Cellectra device. The vaccine targeted a high proportion of neoantigens per patient, resulting in an average of 26.3 neoantigens per patient (range 4–40). The therapy was well tolerated, with no grade 3 or higher TRAEs. Of the 22 patients whose immunology data were available, 19 (86.4%) generated neoantigen-specific T cell responses. The vaccine generated both CD4^+^ and CD8^+^ T cell responses. The authors reported the generation of newer TCR clones as well as the expansion of preexisting T cells, >7-fold in several patients, in response to vaccination. They also demonstrated the migration of newly generated T cells from peripheral blood to tumor with high frequency. The ORR was 30.6% (based on 34/36 evaluable patients), including 3 + 1 surgical CRs, which is almost double the ORR for HCC patients on anti-PD-1/PD-L1 therapy. The median PFS was 4.2 months and the mOS was 19.9 months. The authors also conducted an analysis of circulating tumor DNA (ctDNA) in 13 patients and found that decrease in ctDNA was a strong predictor for OS (*p* = 0.01). The ctDNA levels tracked well with observed CR/PR/SD by imaging for most patients. In two patients who were classified as SD and PR by imaging, the authors observed a ctDNA decrease of 95% and 100%, respectively, highlighting the utility of this analysis for potentially predicting long-term response. The authors observed shrinking of large tumors, as well as elimination of metastatic tumors, demonstrating for the first time that neoantigen vaccines can be effective in shrinking large, established tumors.^136^ In this phase 1/2a study, the mOS was superior to first-line-failure HCC patients treated with pembrolizumab alone (mOS 12.9 months).^147^ This study supports the ability of this neoantigen approach containing a large payload of antigens to impact advanced-stage disease. Further study of this important tool for cold cancers such as HCC is important.

### Nucleic acid platform for personalized cancer vaccines

Post-COVID-19 pandemic, advances in the manufacturing of mRNA-based vaccines and their ability to generate T cell responses have given rise to several neoantigen-targeting vaccines. It is interesting to compare with the above studies as the mRNA platform represents another nucleic acid-based platform that can be used for the manufacture of cancer vaccines, especially personalized vaccines targeting neoantigens. Preclinical studies have demonstrated the feasibility of developing mRNA vaccines targeting neoantigens in animal models of different cancers.^148^^,^^149^ Very recently, four high-profile studies described clinical trial results from the use of nucleic acid vaccines targeting neoantigens in different types of cancers.^136^^,^^140^^,^^150^^,^^151^ The key findings from each study are summarized in Table 2.

**Table 2:** Summary of recent clinical trials using nucleic acid vaccines against neoantigens for cancer

Attribute	KEYNOTE-942^151^	iNEST^140^	GT-30^136^	SLATEv1^150^
Cancer type	high-risk resected melanoma	PDAC	advanced hepatocellular carcinoma	metastatic solid tumors
Therapy type	mRNA-4157 + pembrolizumab	autogene cevumeran + atezolizumab + chemotherapy	DNA plasmid vaccine (GNOS-PV02) + pembrolizumab	ChAd prime + samRNA booster + ipilimumab + nivolumab
Vaccine adjuvant	none	none	plasmid IL-12	PADRE, tetanus toxoid
Phase	2b	1	1/2	1/2
Vaccine delivery method	IM mRNA	IV lipoplex nanoparticle	ID DNA plasmid with EP	IM for both
Neoantigens in vaccine	average: 33 (range 9–34)	average: 14.5 (range 1–20)	average: 26.3^a^ (range 4–40)	20
Linker	none	GS linker	furin cleavage site	unknown
Time to vaccine delivery	weeks to months	within 9.4 weeks after surgery	within 6.1 weeks after surgery	not reported
AEs	grade ≥3: 25% (combination), 18% (monotherapy); no grade 4–5 for mRNA-4157	grade 3 in 6% of patients (fever, hypertension)	no grade ≥3 TRAEs; common: injection-site reactions	grade 3–4 in 10.5%; common: pyrexia, fatigue, nausea, vomiting
TRAEs leading to withdrawals	29/104 (combination), 7/49 (monotherapy)	none	none	none
T cell responses	T cell responses not mentioned	CD8^+^: strong polyfunctional responses in 50% of patients; CD4^+^: detected but less functional than CD8^+^ responses	CD8^+^: robust clonal expansion, cytotoxicity, and tumor infiltration; CD4^+^: present but less proliferative and cytotoxic than CD8^+^ responses; T cell responses observed in 86.4% of patients	observed in 31% of the patientsCD4^+^ vs. CD8^+^ responses not mentioned
Combination therapy outcome	HR for recurrence/death 0.561 (*p* = 0.053); 18-month recurrence-free survival 79% (vs. 62% with monotherapy)	vaccine responders had not reached recurrence median; non-responders 13.4 months (*p* = 0.003)	ORR 30.6%; CR in 8.3% of patients; mPFS not reached at time of publication	ORR 0%; mPFS 1.9 months; mOS 7.9 months

AEs, adverse events; ChAd, chimpanzee-derived adenovirus; GS, glycine-serine; HR, hazard ratio; ID, intradermal; IM, intramuscular; IV, intravenous; mOS, median overall survival; mPFS, median progression-free survival; ORR, overall response rate; PADRE, pan DR epitope; PDAC, pancreatic ductal adenocarcinoma; samRNA, self-amplifying mRNA; TRAEs, treatment-related adverse events.

a From 22 patients for whom immunology data are reported.

These studies demonstrate the progress and challenges in using nucleic acid-based vaccines for targeting cancer neoantigens. The KEYNOTE-942 study highlights the impact of combining mRNA vaccines with anti-PD-1, which reduced the chances of recurrence and improved OS in high-risk melanoma patients.^151^^,^^152^ Detailed characterization of immune responses induced by the vaccine has not yet been reported, but the relationship between this study and the immune responses should be informative to confirm and build on these results.

The iNEST study tested the application of the mRNA neoantigen technology in PDAC patients who suffer from dismal prognoses and desperately need newer therapies.^140^ Overall, they report a 50% response rate in this study of 16 individuals. Fully, 50% of the subjects did not make T cell responses to the vaccine, and these were the same patients who did not show a clinical response. For the immune responders, the median response was against two neoantigens.

Another study by the same group studied mRNA vaccines targeting neoantigens either as a monotherapy or in combination with anti-PD-L1 antibody atezolizumab in pretreated patients with advanced solid tumors (this study was registered at ClinicalTrials.gov: NCT03289962). In an *ex vivo* ELISpot assay without previous *in vitro* expansion, the authors observed T cell responses in 71% of the participants, with no difference in patients who received the vaccine as a monotherapy versus those who received the vaccine in combination with atezolizumab. In the patients who responded, the median number of neoantigens against which an immune response was observed was two. A more detailed characterization of the immune responses was performed in 17 patients where the authors expanded the T cells *in vitro* for 14 days before testing CD4^+^ versus CD8^+^ T cell responses. In this subset of patients, they observed T cell responses against 52% of the neoantigens targeted, 59% of which were targeted by CD4^+^ T cells, 26% were targeted by CD8^+^ T cells, and 15% were targeted by both CD4^+^ and CD8^+^ T cells. In the monotherapy group, 1 in 30 patients had a CR, 12 had SD, and the others had progressive disease (ORR 3.3%). In the combination therapy group (*n* = 183), across different cancer types, the ORR was 8.2%, including 2 patients who experienced a CR.^153^

Targeting large numbers of neoantigens was a feature of the GT-30 plasmid-based trial (HCC patients who had failed first-line therapy), which included an average of 26.3 neoantigens per patient, and the authors observed a correlation between the number of neoantigens included and response rates.^136^ Including more neoantigens in the vaccine construct likely contributed to the impact observed and the demonstration of the vaccine regime to shrink both primary and metastatic lesions in patients, improving clinical responses. This study strongly supports that this DNA vaccine approach was of benefit to the HCC patients, which is traditionally considered an immunologically cold tumor. It will be important to see the follow-up immunology response rates and to continue to follow overall response rates, as many patients remained on study at the time of publication, due to the positive impact of the vaccine.

The SLATEv1 sought to overcome the challenges of manufacturing individual, personalized vaccines for each patient by targeting shared neoantigens across different tumor types. This approach had the additional benefit of mainly targeting driver mutations, which are critical for tumor formation.^150^ In this study, all patients received an initial vaccine targeting 20 shared neoantigens using a chimpanzee-derived adenovirus vector (ChAd68). This was boosted with self-amplifying mRNA (samRNA) encoding for the same 20 neoantigens. The low immunogenicity (only 31% of the patients had any T cell response), despite using the powerful combination of ChAd + samRNA, and lack of clinical response (ORR 0%) in this study suggests that shared neoantigens are more challenging to target compared to personalized neoantigens and that the combined modality used may need enhancement for neoantigen studies. Newer approaches to further improve immune responses or combining these with personalized neoantigen therapy could boost response rates.

## DNA delivery of biologics for cancer

The application of therapeutic antibodies has demonstrated significant improvement in cancer treatment. Technological advancements have accelerated the process of antibody discovery and facilitated the development of novel antibody formats, enhancing their efficacy *in vivo*.^154^^,^^155^ Currently, more than 25 mAbs and 11 bispecific antibodies are approved for the treatment of different types of cancer.^156^^,^^157^ The costs of development and manufacturing of antibodies remain a challenge. In addition, bispecific antibodies tend to have short-term expression *in vivo*, which limits aspects of patient therapies. The strategy of delivering antibodies or bispecifics through directly administering DNA may be a tool to help address some of these challenges.^158^

DNA-encoded mAbs (dmAbs) offer potential as a scalable and cost-effective solution for therapeutic antibody delivery, such as plasmid delivery of ICB. For example, a single IM injection of a plasmid expressing a CTLA4 (ipilimumab) dmAb, followed by EP, achieved peak serum levels of ∼85 μg/mL in mice, with sustained expression exceeding 1 year and serum concentrations remaining above 15 μg/mL. The *in vivo*-produced anti-human CTLA4 dmAb was functional, effectively inducing T cell activation.^159^ In comparison, pharmacokinetic studies of ipilimumab in melanoma patients receiving treatment every 3 weeks demonstrated a mean terminal half-life of 14.7 days and a trough concentration of 21.8 μg/mL, with a 3-mg/kg regimen,^160^ highlighting that DNA delivery of mAbs can reach and maintain therapeutically relevant levels *in vivo* in these small animal studies, potentially being dose sparing. In an additional example, a dmAb targeting human PD-1 was developed and showed expression lasting longer than 4 months *in vivo* with strong binding to human CD4^+^ and CD8^+^ T cells.^161^

In addition to checkpoint inhibitors, dmAbs targeting TAAs have shown anti-tumor effects *in vivo*. For instance, the expression of an HER2-targeting dmAb *in vivo* lasted for over 9 months with a single administration. Treatment with an HER2 dmAb significantly delayed tumor progression in nude mice models of ovarian cancer (OVCAR3) and HER2-overexpressing breast cancer (Brpkp110).^162^

Bispecific immune cell engagers are engineered antibodies that direct immune cells toward tumor cells, with one arm binding to a TAA and another binding an activating or inhibiting receptor expressed on immune cells. The majority of the work in this field focuses on T cell engagers (TCEs) and NK cell engagers (NKCEs).^163^ We summarize some recent work in this area in the following.

Perales-Puchalt et al. developed a DNA-encoded bispecific T cell engager (dBTE) against HER2xCD3. The dBTE expressed for over 100 days *in vivo* and induced HER2-specific OVCAR3 tumor killing in the presence of human T cells *in vitro*. In NSG mice implanted with OVCAR3 tumors, a single-dose administration of HER2 dBTE induced significant tumor regression, with the complete elimination of 8 in 10 tumors.^162^ In another study, a follicle-stimulating hormone receptor (FSHR) targeting mAb was developed that binds specifically to FSHR expressed on the surface of tumor cells. This potent antibody was next converted into a dBTE. The FSHRxCD3 dBTE suppressed FSHR overexpressing K562 and OVCAR3 tumor progression *in vivo*.^164^

The designing of BTEs, including the orientation of heavy and light chains and valency, impacts the specificity and efficacy of dBTEs *in vivo*. The canonical BTE format connects two single-chain variable fragments (scFvs) in tandem with a short linker.^165^ In a study targeting IL13Rα2 for glioblastoma treatment, various variable region heavy (V_H_) and light (V_L_) chain orientations of two distinct scFvs against IL13Rα2 were evaluated. Interestingly, the V_H_-V_L_-V_L_-V_H_ orientation resulted in a high level of cytokine release when cocultured with a cell line lacking target antigen expression, while the V_L_-V_H_-V_H_-V_L_ orientation showed the highest activation specificity, minimizing off-target effects. The study further highlighted that DNA delivery of BTEs significantly extends the duration of *in vivo* expression compared to recombinant BTE delivery. This finding underscores the potential of the DNA platform to overcome the high costs associated with BTE manufacturing and purification, while improving patient accessibility by reducing the frequency of required doses. When IL13Rα2xCD3 dBTE was delivered systemically, it successfully crossed the blood-brain barrier, controlled the growth of orthotopic glioblastoma tumors, and extended survival in treated animals.^112^ Moreover, the combination of dual dBTEs targeting EGFRvIII and HER2 demonstrated enhanced tumor control compared to single-target BTE treatments in an orthotopic glioblastoma model.^114^ To address challenges related to antigen loss, trispecific T cell engagers (TriTEs), which link an anti-CD3 scFv with two additional scFvs targeting distinct TAAs, offer a promising strategy to mitigate tumor escape. In a related glioblastoma study, Park et al. demonstrate that a DNA-encoding TriTE (dTriTE) targeting IL13Rα2 and EGFRvIII achieved long-term survival in treated mice. They used a heterogeneous GBM model by injecting different tumors expressing either just IL13Rα2 or EGFRvIII into the same mouse. Tumor control by dTriTE was significantly better than mice treated with each dBTE individually or with mice treated with both dBTEs simultaneously.^115^

The half-life of canonical recombinant BTEs is relatively short, ranging from 2 to 4 h, necessitating continuous administration to maintain therapeutic levels.^166^ In case of dBTE, *in vivo* expression peaked around 4 days at ∼100 ng/mL, began to decline by day 7, and became undetectable by day 21.^112^ Therefore, frequent administration is still required to maintain BTE levels *in vivo*.^115^ To address this limitation, the BTE constructs have been modified by fusing BTE to an Fc domain, thereby extending the half-life.^167^ For example, a persistent BTE targeting CD19xCD3 exhibited a half-life of 210 h in non-human primate serum, and a CA9xCD3 BTE displayed a half-life of ∼100 h in mouse serum.^166^^,^^168^ However, the canonical BTE format retained a key advantage of superior tumor distribution, probably because of its smaller size. To address this, O’Connell et al. developed a novel format, the persistent multivalent BTE (PMTE), equipped with two anti-CA9 scFvs flanking an anti-CD3 scFv and a human Fc domain. The PMTE format enhanced both tumor penetration and half-life. *In vivo* delivery of the PMTE construct via DNA demonstrated improved tumor infiltration and more effective tumor control compared to both canonical and persistent BTE formats.^168^ The PMTE format was also adapted to a CD45-targeting BTE, which can be a universal BTE against all hematopoietic cancers. This PMTE had similar potency compared to CD45-targeting chimeric antigen receptor T (CAR-T) cells.^169^

NKCEs are designed to bridge NK cells and tumor cells by fusing one scFv targeting a TAA with another scFv targeting an NK cell activating or inhibitory receptor.^170^ Studies have reported that the sialic acid-binding immunoglobulin-like lectin (siglec)-sialic acid interaction can modulate the immune responses in the TME.^171^ Siglec-7 and siglec-9 are receptors expressed on NK cells that interact with sialic acid expressed on tumors and reduce NK cell activity. The cytotoxicity of NK cells is enhanced by the desialylation of tumor cells.^172^^,^^173^ Therefore, designing biologics that block the interactions of siglec-7 or siglec-9 and their ligands can activate NK cell-mediated tumor-killing responses. In one study, a dmAb targeting siglec-7 was administered into mice in an ovarian tumor model (OVISE). This treatment shrunk ovarian tumors and improved the survival of tumor-bearing mice. To improve the specificity of targeting and NK cell responses, a siglec-7xFSHR NKCE was engineered. Tumor treatment with DNA encoding this NKCE demonstrated improved tumor suppression and NSG-K mice survival in the OVCAR3 challenge model.^174^

Biologics that either directly target tumors or redirect both innate and adaptive immunity against tumors can be delivered using DNA technology. Furthermore, IM-delivered biologics are secreted into circulation and can travel to any tumor site, including crossing the blood-brain barrier (Table 3). DNA delivery of biologics has several advantages over recombinant biologics, including simplified delivery and lower costs of therapeutics. Further study of these biologics for translation into the clinic is warranted.

**Table 3:** Selected preclinical studies for DNA-delivered biologics in cancer

Target	Tumor type	Biologic type	Key takeaways	Reference
CTLA4	Several	dmAb	aCTLA4 dmAb has improved pharmacokinetics over recombinant antibody delivery and can control multiple tumor types *in vivo*	Duperret et al.^159^
PD-1		dmAb	improved expression of aPD-1 dmAb and strong binding to CD4 and CD8 T cells	Perales-Puchalt et al.^161^
HER2	ovarian/breast	dmAb	*in vivo*-produced dmAb against HER2 lasts >9 months and controls growth of ovarian and breast tumors	Perales-Puchalt et al.^162^
Siglec-7	ovarian	dmAb	long-term *in vivo* expression of siglec-7 dmAb, tumor control, and enhanced survival of ovarian tumor-bearing mice	Bordoloi et al.^174^
HER2	ovarian	dBTE	HER2xCD3 dBTE lasts for >100 days *in vivo*, controls growth of ovarian tumors	Perales-Puchalt et al.^162^
FSHR	ovarian	dBTE	FSHRxCD3 dBTE induces potent T cell effector functions, controls growth of FSHR^+^ cells *in vivo*	Bordoloi et al.^164^
FSHR	ovarian	dNKCE	FSHRxSiglec7 bispecific NK cell engager improved tumor targeting and controls growth of FSHR-expressing tumors *in vivo*	Bordoloi et al. ^174^
IL13Rα2	GBM	dBTE	highlights impacts of HL chain orientation on BTE specificity and efficacy, demonstrates systemically delivered dBTE can control growth of orthotopic GBM tumors	Pratik et al.^112^
EGFRvIII, HER2	GBM	dBTE	co-treatment with 2 dBTEs (EGFRvIIIxCD3 and HER2xCD3) can overcome Ag heterogeneity/loss in orthotopic GBM tumors	Park et al.^114^
IL13Rα2, EGFRvIII	GBM	dTriTE	trispecific T cell engager activates GBM patient-derived T cells, overcomes Ag heterogeneity in orthotopic GBM	Park et al.^115^
CA9	ccRCC	PMTE	second-generation PMTE against CA9 demonstrates enhanced avidity and pharmacokinetics over first-generation BTE, controls several ccRCC tumors *in vivo*	O’Connell et al.^168^
CD45	multiple hematopoietic cancers	PMTE, CAR-T cell	PMTE and CAR-T cells target only malignant CD45 cells, leaving epitope-edited CD45 cells intact; can be used as universal therapy for blood cancers	Wellhausen et al.^169^

Ag, antigen; CAR-T, chimeric antigen receptor T cell; ccRCC, clear cell renal cell carcinoma; CTLA4, cytotoxic T-lymphocyte associated protein 4; dBTE, DNA-encoded bispecific T cell engager; dmAb, DNA-encoded monoclonal antibody; dNKCE, DNA-encoded NK cell engager; dTriTE, DNA-encoded trispecific T cell engager; FSHR, follicle-stimulating hormone receptor; GBM, glioblastoma multiforme; HER2, human epidermal growth factor receptor 2; NK, natural killer; PD-1, programmed cell death protein-1; PMTE, persistent multivalent bispecific T cell engager.

## Conclusions

Plasmid-encoded DNA immunizations for cancer immunotherapy have made important advances over the past few years. Improvements in the fundamental understanding of tumor immunology and vaccine technology, as well as platform-specific advancements such as EP and other delivery devices, improved formulations, and the use of IM and ID delivery have contributed to improved clinical data generated by these immunization approaches to cancer. EP using the Cellectra device has been used by groups in studies of several different types of cancers and found to be safe, with adverse effects limited to injection site pain and rash that usually resolve within 1 day. The EP causes a temporary increase in membrane permeability for the DNA, leading to enhanced uptake and thus improved immune responses as well as biologic production.^175^

DNA vaccines as a single biologic have been mildly successful, and there is now clinical evidence that the use of cytokine adjuvants can boost cellular and humoral immune responses, leading to improved patient outcomes.^46^^,^^50^^,^^85^^,^^88^ The addition of ICB, especially anti-PD-1/PD-L1, to the vaccine regimen has also enhanced T cell responses and patient outcomes.^48^^,^^113^ DNA vaccines have also generated responses in patients who were previously refractory to anti-PD-1 therapies, allowing them to respond to anti-PD-1, potentially expanding the number of patients who can benefit from these therapies.^121^^,^^124^ The combination of vaccine with ICB also had reduced AEs compared to dual-ICB therapy, highlighting another way in which DNA immunization may benefit cancer patients without compromising efficacy. While most of the ICB studies were done with anti-PD-1/PD-L1 therapies, it will be interesting to study how other ICB therapies such as anti-CTLA4 and anti-Lag3 can combine with DNA vaccines.

Advances in DNA sequencing technologies and *in silico* methods for neoantigen predictions have further contributed to the development of technology supporting personalized nucleic acid mRNA and DNA vaccines. The decreased costs of sequencing and advances in manufacturing of nucleic acids are making it faster and cheaper to manufacture personalized vaccines and have the potential to revolutionize the field of personalized therapy for diverse diseases. The results from different clinical trials by using different forms of nucleic acids, vaccine designs, and adjuvants and across diverse types of cancers further highlight their potential.

The DNA-encoded delivery of other biologics such as mAbs and bispecific/trispecific immune cell engagers demonstrates the importance of their further study and translation to the clinic. A currently running clinical trial evaluating the delivery of dmAbs in humans based on two anti-COVID-19 mAbs (this study was registered at ClinicalTrials.gov: NCT05293249) is important to provide further insights into using the DNA platform for antibody or antibody derivatives such as bispecifics in cancer immunotherapy trials.

DNA vaccines have shown promising outcomes in early-stage clinical trials, indicating the potential for further exploration in larger studies. Notably, they have demonstrated therapeutic effects in patients with various cancer types, including those typically regarded as immunologically cold tumors, such as breast cancer, prostate cancer, neuroblastoma, and HCC. While the growing body of evidence underscores their potential in cancer treatment, additional research is needed to fully understand their efficacy and optimize their application in diverse clinical settings.

## Acknowledgments

We would like to thank The Wistar Institute’s core facilities for help with the manuscript. Illustrations in the manuscript were made using BioRender. 10.13039/100021318D.B.W. is supported in part by the W.W. Smith Charitable Trust Professorship in Cancer Research, The Jill and Mark Fishman Foundation, Inovio Pharmaceuticals SRA and Geneos Therapeutics SRA.

## Author contributions

D.B.W. and P.S.B. conceptualized the study. P.S.B. did the initial research and analysis. P.S.B., J.J., and S.Z. wrote the initial draft of the manuscript, with guidance from and discussion with D.B.W. D.B.W. and P.S.B. performed the review and editing. All authors have read and approved the article.

## Declaration of interests

D.B.W. has received a commercial research grant from Inovio Pharmaceuticals; has received speakers’ bureau honoraria from Inovio Pharmaceuticals, AstraZeneca, and Pfizer; has ownership interest (including patents) in Inovio Pharmaceuticals; and is a consultant/advisory board member for Inovio Pharmaceuticals, Geneos, Advaccine, Sanofi, and Sumitomo Dainippon Pharma.
